# Challenges to achieving low palatal fistula rates following primary cleft palate repair: experience of an institution in Uganda

**DOI:** 10.1186/s13104-018-3459-6

**Published:** 2018-06-07

**Authors:** Josephine Linda Katusabe, Andrew Hodges, George William Galiwango, Edgar M. Mulogo

**Affiliations:** 1 Comprehensive Rehabilitation Services Uganda (CoRSU) Hospital, P.O.Box 46, Kisubi, Uganda; 2 Mbarara University of Science and Technology, P.O.Box 1410, Mbarara, Uganda

**Keywords:** Cleft palate, Primary palate repair, Palatal fistula

## Abstract

**Objective:**

To determine frequency of palatal fistula following primary cleft palate repair and the associated factors as a measure of cleft palate repair outcome and its challenges at a cleft centre in Uganda.

**Results:**

Between May and December 2016, 54 children with cleft palate were followed up at Comprehensive Rehabilitation services of Uganda (CoRSU) hospital, from time of primary cleft palate repair until at least 3 months postoperative to determine whether they developed palatal fistula or not. Frequency of palatal fistula was 35%. Factors associated with increased fistula formation were cleft width wider than 12 mm (p = 0.006), palatal index greater than 0.4 (p = 0.046), presence of malnutrition at initial outpatient assessment (p = 0.0057) and at time of surgery (p = 0.008), two-stage palate repair (p = 0.005) and postoperative infection (p = 0.003). Severe clefting (palatal index greater than 0.4) was seen in 74% of patients and malnutrition (Low weight for age) seen in 48% of patients. Palatal fistula rates at our institution were high compared to reports in literature. The high proportions of severe clefting and malnutrition observed in our population that was also poor and unable to afford feeding supplements increased likelihood of fistula formation and posed challenges to achieving low fistula rates in our setting.

**Electronic supplementary material:**

The online version of this article (10.1186/s13104-018-3459-6) contains supplementary material, which is available to authorized users.

## Introduction

Palatal fistula is a failure of healing or breakdown in the primary surgical repair of a cleft palate [1]. Palatal fistula results in persistent communication between oral and nasal cavities leading to unpleasant symptoms such as nasal spillage of feeds, hypernasal speech, articulation problems which undermine the success of palate repair [2]. A low incidence of palatal fistula is one of the indicators of successful cleft palate repair [3].

Incidence of palatal fistula in literature ranges from 0 to 35% [1, 3–8] with overall incidence of 8.6% reported by a meta-analysis of studies in Europe, America, Asia and Africa [9]. Risk factors of palatal fistula reported include type of cleft, cleft palate width, surgeon’s experience, timing and technique of repair. There is a paucity of studies in Africa and Uganda assessing frequency of palatal fistula and associated factors following cleft palate repair. In Uganda, reports show that most children with cleft palate are already malnourished when they first present to hospital and may continue failing to thrive if no timely intervention is done [10–12]. Effect of this malnutrition on surgical outcome of palate repair has not been studied.

Our study aimed to determine frequency of palatal fistula following primary palate repair and the associated factors at CoRSU hospital in order to assess our cleft palate repair outcome and also establish the challenges to achieving low fistula rates.

## Main text

### Methods

A prospective case series was conducted from May to December 2016 at CoRSU hospital, a specialized hospital in Uganda offering free cleft palate surgery. Children with unrepaired cleft palate, whose caregivers gave written consent to participate in the study were enrolled and followed up from time of primary cleft palate repair until at least 3 months postoperative to determine whether they developed palatal fistula or not. Primary palate repair (surgery on cleft palates that have not been repaired before) was performed either as single-stage repair of both hard and soft palate or as a two-stage procedure involving hard palate repair with vomer flap in first stage and soft palate repair 3 months later in second stage. Surgical techniques included intravelar veloplasty for soft palate, Von Langenbeck flaps, Bardarch flaps and Hybrid flaps for hard palate (see Additional file 1 showing description of surgical techniques).

Desired perioperative information including age, weight and length, type of cleft, type of surgery, surgical technique and surgeon’s experience (based on volume of palate surgeries performed annually) was recorded in pretested data forms. Preoperative dental casts of each palate were made, from which cleft width and palatal shelf widths were measured using Castroviejo calipers (see Additional file 2 showing dimensions measured). Weight for length and weight for age z-scores were calculated and compared with W.H.O reference values to determine nutrition status. At postoperative review, a consultant plastic surgeon inspected the palate using a clinical torch and tongue depressor to determine presence or absence of fistulas. Only fistulas posterior to incisive foramen were considered. Statistical analysis of data was done using STATA version 12.0. Chi square test, student t test, and multivariate logistic regression were performed to determine factors associated with palatal fistula. Probability values (p-values) less than 0.05 were considered statistically significant.

### Results

A total of 78 children were enrolled but only 54 patients followed up at CoRSU hospital were analysed. Median age was 6 months (range 3–192 months) with 61% of participants below 6 months. Female to male ratio was 1.1:1. Unilateral cleft lip and palate was seen in 52%, bilateral cleft lip and palate in 37% and isolated cleft palate in 11% of patients. Most of the caregivers (61%) resided in rural settings with median monthly income of 28 US dollars. Forty-eight percent (48%) of patients had low weight for length at initial outpatient assessment and 62% of these received nutrition rehabilitation before surgery. Median weight at surgery was 5.7 kg (range 3.5–50 kg). Twenty-four percent (24%) were underweight (low weight for age) and 15% were severely underweight at time of surgery despite attaining minimum required weight for surgery (3.5 kg). Mean cleft width was 12 mm with 48% of patients having clefts wider than 12 mm. Mean ratio of cleft width to sum of palatal shelf width (palatal index) was 0.4 with 74% of patients having palatal index greater than 0.4.

Single-stage repair was done in 67% of patients while 33% had two-stage repair. Vomer flap dehiscence occurred in 67% of two-stage repairs and required hard palate re-repair at second stage. Large-volume operator (surgeon performing over 50 palate surgeries annually) performed 67%, low-volume operator (surgeon performing less than 50 palate surgeries annually) performed 24% and trainee surgeon performed 9% of the surgeries. Von Langenbeck flaps were used in 52%, Bardarch in 22% and hybrid in 26% of patients.

Postoperative complications included early postoperative infection estimated as persistent fever with leukocytosis requiring antibiotics (56%), difficulty in breathing requiring oxygen (38%) and flap dehiscence (6%). 37% of participants reported late postoperative infection estimated as falling sick within 4 weeks after discharge and 60% of these required admission to a hospital near home. Average follow up period was 6 months.

Overall frequency of palatal fistula was 35% (95% CI 22.4, 47.9) with 19 out of 54 patients developing palatal fistula. Twenty-eight percent (15 out of 54) had fistulas requiring surgical repair. Hard palate (Pittsburgh IV) was involved in 58%, Soft palate (Pittsburgh II) involved in 25% and junction of hard and soft palate (Pittsburgh III) involved in 17% of fistula. (See Additional file 2 showing a case with hard palate fistula).

Factors associated with increased fistula formation were: Low weight for length at initial outpatient assessment (p = 0.0057), low weight for age at time of surgery (p = 0.008), cleft width wider than 12 mm (p = 0.006), palatal index greater than 0.4 (p = 0.046), two-stage palate repair (p = 0.005), early postoperative infection (p = 0.003) and late postoperative infection (p = 0.0004) as shown in Tables 1, 2 and 3. Cleft width was an independent predictor of palatal fistula at multivariate analysis (p = 0.03, Adjusted odds ratio = 4.4). No association was found between age, surgical technique, surgeon’s experience and fistula formation.

**Table 1:** showing preoperative factors associated with palatal fistula

Preoperative factor	No fistula (%)	Fistula present (%)	COR (95% CI)	χ^2^ p-value
Age of the child				0.614
0–6 months	21 (60)	12 (63)	1	
7–12 months	9 (26)	6 (32)	1.2 (0.3–4.1)	
13–24 months	2 (6)	1 (5)	0.9 (0.1–10.7)	
> 24 months	3 (9)	0 (0)	–	
Sex				0.293
Male	15 (43)	11 (58)	1	
Female	20 (57)	8 (42)	0.5 (0.2–1.7)	
Type of cleft deformity				0.574
Isolated cleft palate	5 (14)	1 (5)	1	
Unilateral cleft lip and palate	18 (51)	10 (53)	2.8 (0.3–27.2)	
Bilateral cleft lip and palate	12 (34)	8 (42)	3.3 (0.3–34.1)	
Weigh for length at initial outpatient assessment				**0.0057**
No malnutrition	23 (66)	5 (26)	1	
Malnutrition	12 (34)	14 (74)	5. 4 (1.6–18.5)	
Weight at time of surgery				0.224
< 5 kg	6 (17)	6 (32)	1	
≥ 5 kg	29 (83)	13 (68)	0. 4 (0.12–1.6)	
Weight for age at time of surgery				**0.008**
Not underweight	21 (60)	5 (26)	1	
Under-weight	9 (26)	4 (21)	1.9 (0.4–8.6)	
Severely underweight	5 (14)	10 (53)	8.4 (2.0–35.8)	
Cleft width at initial surgery				**0.006**
< 12 mm	23 (72)	5 (26)	1	
≥ 12 mm	12 (28)	14 (74)	5.6 (1.6–19.2)	

*COR* denotes Crude odds ratio (unadjusted), *CI* denotes confidence interval, χ^2^ denotes Pearson Chi square, p-values in bold = statistically significant

**Table 2:** Palatal dimensions associated with palatal fistula

Palatal dimension measured	No fistula	Fistula	p-value for t test
	Mean (SD)	Mean (SD)	
Palatal index at initial surgery			
< 0.4 mm	0.32 (0.06)	0.26 (0.09)	0.198
≥ 0.4 mm	0.51 (0.06)	0.58 (0.15)	**0.046**
Palatal index at second stage			
< 0.4 mm	0.28 (0.05)	0.36 (0.03)	0.034
≥ 0.4 mm	0.44 (0.02)	0.51 (0.08)	**0.015**

p-values in bold are statistically significant

**Table 3:** Intraoperative and Postoperative factors associated with palatal fistula

Postoperative factor	No fistula (%)	Fistula present (%)	COR (95% CI)	χ^2^ p-value
Type of palate repair				**0.005**
Single stage	28 (80)	8 (42)	1	
Two stage	7 (20)	11 (58)	5.5 (1.6–18.8)	
Lead surgeon’s experience				0.920
Trainee	3 (8)	2 (11)	1	
Large volume operator	24 (69)	12 (63)	0.75 (0.1–5.1)	
Low volume operator	8 (23)	5 (26)	0.9 (0.1–7.7)	
Technique used for hard palate repair				0.857
Bilateral Von-langenbeck	12 (34)	8 (42)	1	
Bilateral Bardarch	9 (26)	3 (16)	0.5 (0.1–2.4)	
Hybrid flaps	9 (26)	5 (26)	0.8 (0.2–3.4)	
Others	5 (14)	3 (16)	0.9 (0.2–4.9)	
Early postoperative infection (persistent fevers requiring antibiotics)				**0.003**
No	33 (94)	12 (63)	1	
Yes	2 (6)	7 (37)	9.6 (1.8–52.9)	
Late postoperative infection (fell sick within 4 weeks after discharge following surgery)				**0.0004**
No	28 (80)	6 (32)	1	
Yes	7 (20)	13 (68)	8.7 (2.4–31.0)	
Type of feeds given within 3 weeks after surgery				0.658
Unmashed	3 (9)	1 (5)	1	
Liquid or mashed	32 (91)	18 (95)	1.6 (0.2–17.4)	

p-values in bold are statistically significant

*COR* denotes Crude odds ratio (unadjusted), *CI* denotes confidence interval, χ^2^ denotes Pearson Chi square

### Discussion

Frequency of palatal fistula at our institution is high compared to reports in literature [1–9, 13–16] and loss to follow up, difference in population characteristics could be responsible for this discrepancy. Loss to follow up could have resulted in artificially higher fistula rates if some of the patients lost actually had no fistulas. Patients without palatal fistulas don’t experience the related unpleasant symptoms that would otherwise prompt seeking of further treatment and follow up. Loss to follow up could also have been due to low socioeconomic status where most of the caregivers were poor (earning 28 US dollars a month), lived in remote areas and probably could not afford transport to hospital for review. This possibly hindered return of both patients with and without fistulas resulting in under or overestimation of our fistula rates.

The proportion of malnutrition in our population was high, both at initial outpatient assessment and at time of surgery and this probably contributed to the high fistula rates. Results actually showed that low weight for length at initial outpatient assessment and low weight for age at time of surgery were associated with increased fistula formation (Table 1). Malnutrition impedes processes that allow progression of wound healing and has been related to decreased wound tensile strength and increased infection rates [17]. Patients with malnutrition are therefore prone to wound breakdown following surgery which explains their increased likelihood for fistula formation.

Prevalence of malnutrition among children with cleft palate in Africa has been reported to be high by other studies [11, 12, 18, 19]. The trend is that most children with cleft palate present with chronic malnutrition and stunting and many are hypothesized to die before surgery due to malnutrition [10]. Intervention through nutritional rehabilitation and surgery has been recommended to improve their survival [11]. Chronic malnutrition with stunting requires prolonged periods of nutritional rehabilitation before optimal nutrition status can be attained but majority of our patients are poor and cannot afford feeding supplements. This creates a dilemma for the cleft surgeon trying to achieve good surgical outcomes in a setting where optimizing nutrition status before surgery is difficult. During the study period, our centre performed palate repair as early as 3 months, at minimum weight of 3.5 kg, with the aim of improving feeding. With this protocol, some of the children were still underweight at surgery yet it hasn’t been demonstrated that surgery in underweight children with cleft palate actually improves their nutrition status hence there is need for more research.

Palatal index greater than 0.4 is classified as severe clefting with significant tissue deficiency [7]. A high proportion of patients had clefts wider than 12 mm (48%) and palatal index greater than 0.4 (74%) indicating that most of our study population had wide and severe clefting, that was actually found to be associated with increased fistula formation(Tables 1 and 2). Other studies have also shown that cleft width wider than 13–15 mm and palatal index are associated with increased fistula formation [3, 6–8]. According to Parwaz et al., the risk of fistula formation increases as palatal index increases to 0.48 [6]. Wide clefts are prone to tension on closure and are related with more technical difficulties to close than narrow clefts which explains why patients with wide clefts are more likely to develop fistulas [16].

Two-stage palate repair was associated with increased fistula formation in our study (Table 3). This is contrary to other studies that reported significant reduction in cleft width, operating time and fistula formation following early hard palate repair using vomer flap [4, 20–22]. The high rate of vomer flap dehiscence possibly contributed to the increased fistula formation in the two-stage group. Vomer flap dehiscence required re-repair of hard palate at second stage using previously scarred and less pliable vomer tissue that was of poor quality and prone to breakdown. This issue was also raised by Deshpande et al. who found that failed vomer flaps increased risk of fistula formation in subsequent palate repairs [23]. More research is needed to investigate causes of vomer flap failure in our setting which if addressed could help reduce fistula formation. Our centre performs vomer flaps for clefts deemed too wide to close in a single stage, to help reduce cleft width while also providing an intact hard palate to improve suckling. Effectiveness of this two-stage protocol in reducing cleft width and improving nutrition status in our patients with severe clefting and malnutrition needs to be studied to help justify its use in treating wide clefts.

Early and late postoperative infection was a common complication in our study and was associated with palatal fistula formation (Table 3). Postoperative infection is one of the likely reasons for fistula formation but is unlikely in babies unless compromised immunologically or nutritionally [24]. The high proportion of malnutrition seen in our population that mainly comprised babies below 6 months could have predisposed to increased postoperative infection which probably translated into higher fistula rates.

### Conclusion

Frequency of palatal fistula at our institution was high. The high proportions of severe clefting and malnutrition in our population that was also poor and unable to afford feeding supplements increased likelihood of fistula formation and posed challenges to achieving low fistula rates in our setting. We recommend more efforts on optimizing nutrition status before surgery through nutritional education and feeds supplementation, even in the face of a challenging low socioeconomic status. More research is needed to determine effect of surgery on nutrition status of children with cleft palate which will help to guide better the timing of surgery with regard to nutrition status. Further research is also needed to investigate effectiveness of vomer flap in reducing cleft width and improving nutrition status to help justify its benefit in treating wide clefts in our population.

## Limitations

- Lack of funds hindered return of some participants for review resulting in loss to follow up which could have resulted in over or underestimation of fistula rates.
- Study was done at a single surgical centre in Uganda with a small sample size which may not adequately represent palate repair outcome in other Ugandan cleft centres.

## Additional files

**Additional file 1.** **Description of surgical techniques used for primary palate repair in our study.** Brief descriptions of the surgical techniques used for palate repair is provided. These include, Von Langenbeck flap, Bardach flap, Hybrid flap and vomer flap techniques for hard palate repair and intravelar veloplasty for soft palate repair.

**Additional file 2.** **A Figure showing a hard palate fistula and dimensions of the cleft palate that were measured. Figure 1a**) shows a case with a hard palate fistula (Pittsburgh IV**)**. In **Figure 1b**) distance B-C is cleft width measured at junction of hard and soft palate, A-B and C-D are the right and left palatal shelf widths measured at level of the maxillary tuberosities, respectively.

## Abbreviations

CoRSU: comprehensive Rehabilitation Services in Uganda
WHO: World Health Organization
