# Supplementary material for: Challenges to achieving low palatal fistula rates following primary cleft palate repair: experience of an institution in Uganda
Source: BMC Res Notes. 2018 Jun 7;11:358. doi: 10.1186/s13104-018-3459-6 (PMC5992877; doi:10.1186/s13104-018-3459-6)
Supplement: Supplementary file 1 — Additional file 1. Description of surgical techniques used for primary palate repair in our study. Brief descriptions of the surgical techniques used for palate repair is provided. These include, Von Langenbeck flap, Bardach flap, Hybrid flap and vomer flap techniques for hard palate repair and intravelar veloplasty for soft palate repair. [file 13104_2018_3459_MOESM1_ESM.docx]

## TITLE OF ARTICLE:

# CHALLENGES TO ACHIEVING LOW PALATAL FISTULA RATES FOLLOWING PRIMARY CLEFT PALATE REPAIR: EXPERIENCE OF AN INSTITUTION IN UGANDA

# ADDITIONAL FILE 1: Description of surgical techniques used for primary palate repair in our study

Primary cleft palate repair was defined as the surgical repair of a cleft palate that had not been repaired before. It was performed either as single-stage or two- stage procedure. The single -stage palate repair involved repair of both hard and soft palate in one operation. The two-stage palate repair involved repair of the hard palate in the first stage and soft palate repair in the second stage. First stage was performed simultaneously with clef lip repair while second stage was performed 3 months after first stage. Surgical techniques used for hard palate repair included Von Langenbeck flaps, Bardarch flaps, Hybrid flaps and vomer flaps. Intravelar veloplasty was used for all soft palate repairs.

- **Von Langenbeck flap technique**

This technique involved use of two bipedicled mucoperiosteal flaps that were mobilized from the palatal shelves through 2 lateral releasing incisions and approximated in the midline to close cleft in the hard palate. The flaps were based both anteriorly at level of the incisive foramen and posteriorly based on the greater palatine vessels.

- **Bardach flap technique**

This technique, also known as the two flap palatoplasty involved use of two unipedicled mucoperiosteal flaps that were mobilized from the palatal shelves through 2 lateral releasing incisions and approximated in the midline to close cleft in the hard palate. Unlike the Von Langenbeck flaps, Bardach flaps were completely detached anteriorly and based only on the greater palatine vessels.

- **Hybrid flap technique**

This technique involved use of a combination of Von Langenbeck and Bardarch flaps. In this technique, a bipedicled mucoperiosteal flap was raised from the greater segment of the palatal shelves while a unipedicled mucoperiosteal flap was raised from the lesser segment and the two flaps were approximated in midline to close the cleft.

- **Intravelar veloplasty**

This technique of soft palate repair involved dissection of the palatal muscles from their abnormal attachment to posterior edges of the palatal shelves and from the nasal layer. Transverse retro-positioning of the muscles and suturing in midline was then done to restore the levator sling. The soft palate was repaired in 3 layers including nasal mucosa, muscle layer and oral mucosa.

- **Vomer flap technique**

This technique of hard palate repair was used for clefts deemed too wise to close in a single stage without tension. The technique involved use of a mucoperiosteal flap that was raised from the vomer and tucked under the elevated margin of the oral mucosa on the opposite palatal shelf. The raw surfaces of the exposed nasal septum and flap were left to heal by epithelialization. Soft palate repair was delayed until 3 months later and performed as second stage.
