# Supplementary material for: Challenges to achieving low palatal fistula rates following primary cleft palate repair: experience of an institution in Uganda
Source: BMC Res Notes. 2018 Jun 7;11:358. doi: 10.1186/s13104-018-3459-6 (PMC5992877; doi:10.1186/s13104-018-3459-6)
Supplement: Supplementary file 2 — Additional file 2. A Figure showing a hard palate fistula and dimensions of the cleft palate that were measured. Figure 1a) shows a case with a hard palate fistula (Pittsburgh IV). In Figure 1b) distance B-C is cleft width measured at junction of hard and soft palate, A-B and C-D are the right and left palatal shelf widths measured at level of the maxillary tuberosities, respectively. [file 13104_2018_3459_MOESM2_ESM.docx]

## TITLE OF ARTICLE:

# CHALLENGES TO ACHIEVING LOW PALATAL FISTULA RATES FOLLOWING PRIMARY CLEFT PALATE REPAIR: EXPERIENCE OF AN INSTITUTION IN UGANDA

**ADDITIONAL FILE 2: A Figure showing a hard palate fistula and dimensions of the cleft palate measured**


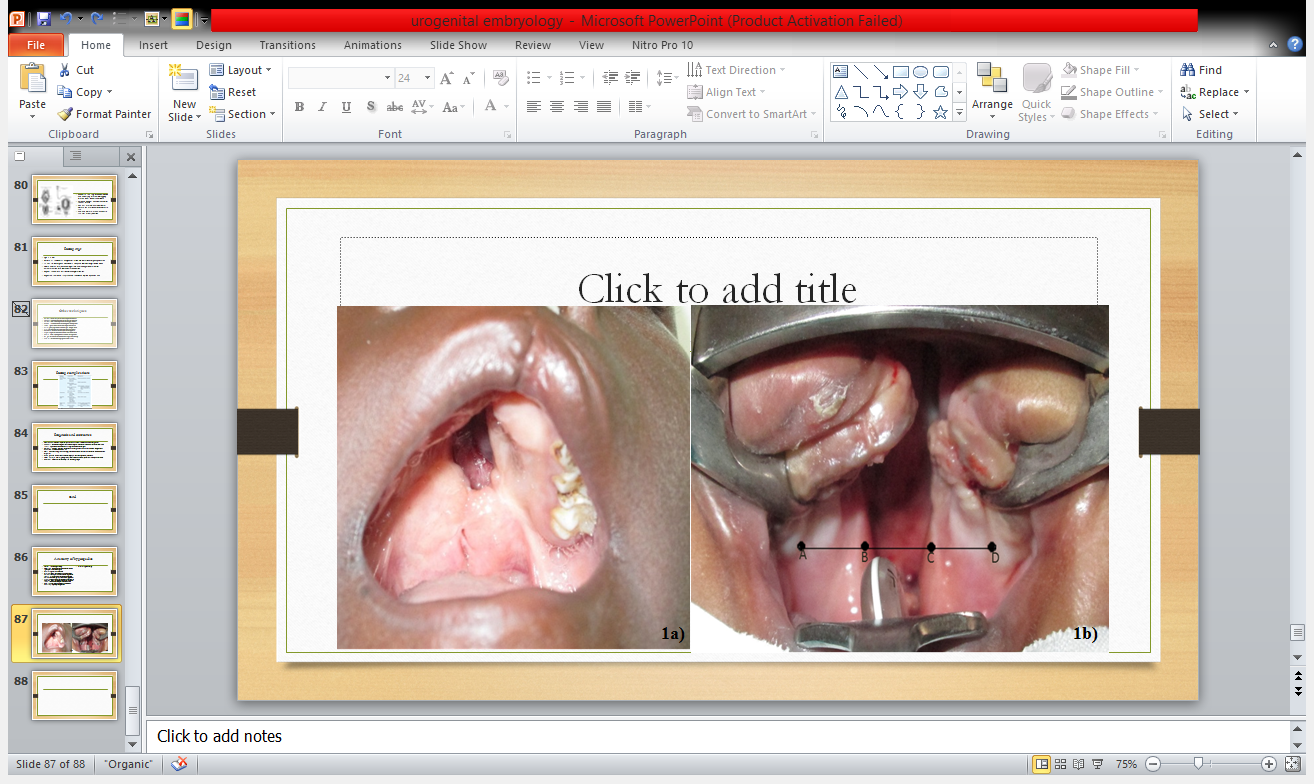


Figure 1a) shows a case with a hard palate fistula (Pittsburgh IV). In Figure 1b) distance B-C is cleft width measured at junction of hard and soft palate, A-B and C-D are the right and left palatal shelf widths measured at level of the maxillary tuberosities, respectively
